# Quercetin-Crosslinked Chitosan Films for Controlled Release of Antimicrobial Drugs 

**DOI:** 10.3389/fbioe.2022.814162

**Published:** 2022-03-14

**Authors:** Helton José Wiggers, Pascale Chevallier, Francesco Copes, Fernanda Heloisa Simch, Felipe da Silva Veloso, Giovana Maria Genevro, Diego Mantovani

**Affiliations:** ^1^ Laboratory for Biomaterials and Bioengineering (LBB-BPK), Associação de Ensino, Pesquisa e Extensão BIOPARK, Toledo, Brazil; ^2^ Laboratory for Biomaterials and Bioengineering (LBB-UL), Canada Research Chair Tier I, Department of Min-Met-Materials Engineering and CHU de Quebec Research Center, Division Regenerative Medicine, Laval University, Quebec, Canada

**Keywords:** chitosan, quercetin, trimethoprim, controlled drug release, antibacterial

## Abstract

Natural polymer-based films, due to their favorable biological and mechanical properties, have demonstrated great potential as coatings for biomedical applications. Among them, chitosan films have been widely studied both as coating materials and as controlled drug release systems. Crosslinkers are often used to tune chitosan’s crosslinking degree and thus to control the drug release kinetics. For this purpose, quercetin, a plant-derived natural polyphenol, has gained attention as a crosslinker, mainly for its intrinsic anti-inflammatory, antioxidant, and antibacterial features. In this study, chitosan films crosslinked with three different concentrations of quercetin (10, 20, and 30% w/w) have been used as controlled release systems for the delivery of the antibacterial drug trimethoprim (TMP, 10% w/w). Physicochemical and antimicrobial properties were investigated. Surface wettability and composition of the films were assessed by contact angle measurements, X-ray photoelectron spectroscopy (XPS), and Fourier-transform infrared spectroscopy (FTIR), respectively. The release kinetic of TMP in phosphate-buffered saline (PBS) and 2-(N-morpholino) ethanesulfonic acid (MES) was studied over time. Finally, antibacterial properties were assessed on *E. coli* and S. *aureus* through Kirby–Bauer disc diffusion and micro-dilution broth assays. Results show that quercetin, at the tested concentrations, clearly increases the crosslinking degree in a dose-dependent manner, thus influencing the release kinetic of the loaded TMP while maintaining its bactericidal effects. In conclusion, this work demonstrates that quercetin-crosslinked chitosan films represent a promising strategy for the design of antibiotic-releasing coatings for biomedical applications.

## 1 Introduction

Healthcare-associated infections (HAIs) are defined as all infections occurring in patients while receiving treatment for other conditions. According to the Center for Diseases Control and Prevention (CDC), HAIs account for an estimated 1.7 million infections and 99,000 associated deaths each year in the United States alone (Healthcare-Associated Infections, 2021). Statistics show that 1 of every 25 patients admitted to a hospital will acquire an HAI, leading to longer hospitalization, readmissions, and, in a worst-case scenario, death. The estimated direct medical cost of HAIs exceeds $30 billion annually in the United States alone (Scott 2009; ODPHP 2021) and $3.4 million per year in expenditures reported by the Canadian government (Health Canada 2018). Among the HAIs, biomaterial-associated infections (BAIs) account for more than half of the total infections (Van Epps and Younger 2016). The use of medical devices (such as catheters and implants) is hampered by biofilm-forming microorganisms’ high rate of colonization of their surfaces.

To prevent BAIs, especially for indwelling medical devices such as central venous and urinary catheters, the introduction of central line bundles (including hand hygiene, maximal barrier precautions, chlorhexidine skin antisepsis, optimal catheter site selection, and daily review of the central line necessity) has been demonstrated to be crucial (Dumont and Nesselrodt 2012; Arrieta et al., 2019). However, they are not sufficient to avoid the insurgence of these infections, and therefore, several new strategies have been reported. Among them, coatings with antibacterial properties have been proposed to limit the colonization and proliferation of infectious bacteria and the subsequent biofilm formation (Li et al., 2021; Stærk et al., 2021). Specifically, drug-eluting or controlled drug release coatings gained interest from researchers. The development of coatings capable of releasing antibacterial compounds at the infection site for a sustained period represents an interesting approach to address the issue. The local delivery of antibacterial agents offers the advantage of a higher concentration at the wound site as compared to systemic delivery, avoiding harmful secondary effects (Stærk et al., 2021). Moreover, the possibility to modulate over time the presence of effective and sustainable concentrations of the antibacterial agents at the site of interest is highly promising (Viola et al., 2017). In the development of drug delivery coatings, the choice of the material is primordial. Polymers, especially biopolymers, represent a viable solution for the development of such coatings (Rebelo et al., 2017).

Biopolymers (also called natural polymers) are produced mainly by living organisms and present, due to their natural origins, favourable characteristics such as biocompatibility, biodegradability, non-cytotoxicity, good availability, and many other beneficial properties (Song et al., 2020). Chitosan is a versatile biopolymer derived from the deacetylation of natural chitin. It is a polysaccharide with positively-charged native amine groups. Its bioactive, antimicrobial, anti-inflammatory, and mucoadhesive properties have been widely studied and demonstrated (Kean and Thanou 2010; Kumar and Kumar 2020). Chitosan has already been used to develop drug-releasing systems (Bernkop-Schnürch and Dünnhaupt 2012). The main drug-releasing mechanism from chitosan matrices involves diffusion and swelling (Younis et al., 2018). However, chitosan films have demonstrated poor stability in aqueous solutions (Park et al., 2002), hindering the release of loaded drugs. Therefore, the controlled release of the loaded drugs has not always been optimal, resulting in a burst release and, consequently, a loss of efficacy over time. Different approaches have been developed to address these issues. One of those involves the use of crosslinkers, molecules containing reactive groups capable of binding to specific functional groups on polymeric molecules. Different crosslinkers have been reported for chitosan, resulting in better mechanical properties, reduced swelling behavior, and hydrophilicity (Nataraj et al., 2018; Khouri et al., 2019). Moreover, the use of crosslinker in chitosan-based materials improved the controlled release of loaded drugs (Tomaz et al., 2018). The main crosslinkers used are genipin, glutaraldehyde, epichlorohydrin, citric acid, and pentasodium tripolyphosphate (TPP). The first three induces covalent chemical bonds, whereas the last two leads to ionic interactions. Despite having some advantages, crosslinking based on ionic interactions remains less valuable than covalent ones: lack of stability and the strong dependence on the medium (pH, salts present, etc.) are the major drawbacks. On the other hand, glutaraldehyde and epichlorohydrin, organic products, have been demonstrated to induce slight cytotoxicity. In this sense, natural crosslinkers are more attractive and widely investigated thanks to their favorable properties. Among them, phenolic compounds, particularly catechols, have gained interest in recent years for their crosslinking behavior and inherent biological properties. For instance, quercetin, a flavanol [quercetin (3,30,40,5,7-pentahydroxyflavone)] commonly found in many seeds, fruits, and vegetables, presents strong antioxidant, anti-inflammatory, and bactericidal properties and low toxicity (Harwood et al., 2007; Aljadaan et al., 2020) and have already been used as a crosslinking agent in the development of chitosan-based materials (Souza et al., 2015; Greco et al., 2018; Bai et al., 2019). However, to our best knowledge, this approach has never been used as a drug carrier for release control.

In this study, the potential of drug-releasing capability of quercetin-crosslinked chitosan films has been investigated. To achieve the desired antibacterial effects for BAI prevention, the developed films have been loaded with trimethoprim (TMP), a synthetic antibiotic that binds with the enzyme dihydrofolate reductase (DHFR), inhibiting the folic acid synthesis pathway (Wróbel et al., 2020). Moreover, the TMP chemical structure presents two amino groups that are able to interact with the hydroxyl groups found on quercetin and chitosan structures resulting in hydrogen bonds that could further help in sustaining TMP release from the chitosan film. Different quercetin concentrations have been tested to produce TMP-loaded chitosan films. The obtained films have been characterized by their physicochemical and antibacterial properties due to TMP release.

## 2 Materials and Methods

### 2.1 Materials

Chitosan (CS, Sigma, medium molecular weight, China), quercetin (Q, Sigma, India), trimethoprim (TMP, Haishing Co. Pte. Ltd., China), acetic acid (HA, Synth, Brazil), phosphate-buffered saline (PBS, Sigma, United Kingdom), sodium hydroxide (NaOH, Sigma, Sweden), acetonitrile (ACN, Merck, Germany), Mueller–Hinton agar (MHA, Kasvy, Spain), Mueller–Hinton broth (MHB, Difco, United States), *Staphylococcus aureus* ATCC 6538 (Lab-Elite™, Brazil), *Escherichia coli* ATCC 8937 (Lab-Elite™, Brazil), 2-(N-morpholino) ethane sulfonic acid (MES, Sigma, United States), triethylamine (TEA, Êxodo Científica, Brazil), and glycerol (Anastacio chemical industry, Brazil) were used in this study.

### 2.2 Chitosan Film Preparation

First, 1.5% m/v chitosan solution was prepared by dissolving chitosan in 1.0% v/v aqueous acetic acid solution under vigorous stirring overnight (ON). The chitosan solution was then filtered using a porous Buchner funnel and a vacuum pump and stored at 4°C until use. A total of 10 mg/ml quercetin solution was prepared by dissolving quercetin in 0.1 M sodium hydroxide. Finally, 10 mg/ml trimethoprim solution was prepared by dissolving the antibiotic in 1% v/v acetic acid solution. Different films were obtained using a casting method. Aliquots from the stock solutions were taken, as described in Table 1 and then poured into 90-mm Petri dishes and finally incubated at 37 °C to allow the solvent evaporation until a constant mass was reached. The obtained quercetin-crosslinked chitosan films showed thicknesses varying from 20 to 25 µm.

**TABLE 1:** Chitosan–quercetin film formulation and synthesis.

Blank films				
Film	Chitosan (ml)	Quercetin (ml)	TMP (ml)	Water (ml)
CS	6.67	0.0	0.0	3.33
CS/Q10	6.67	1.0	0.0	2.33
CS/Q20	6.67	2.0	0.0	1.33
CS/Q30	6.67	3.0	0.0	0.33
**TMP-loaded films**				
**Film**	**Chitosan (ml)**	**Quercetin (ml)**	**TMP (ml)**	**Water (ml)**
CS/TMP	6.67	0.0	1.0	2.33
CS/Q10/TMP	6.67	1.0	1.0	1.33
CS/Q20/TMP	6.67	2.0	1.0	0.33
CS/Q30/TMP	6.67	3.0	1.0	0.0

Volumes refer to the stock solutions.

### 2.3 Surface Characterization

The surface wettability and composition of the films were assessed by contact angle measurements, X-ray photoelectron spectroscopy (XPS), and Fourier-transform infrared (FTIR) spectroscopy, respectively.

Static contact angle measurements were obtained using a VCA 2500 XE system (AST^®^ Billeria, MA, United States). The analyses were performed at room temperature, with 1 μL ultrapure water droplets applied in three different regions per sample.

XPS analyses were carried out using Physical Electronics PHI 5600-ci equipment (MN, United States). A standard aluminum X-ray source (1,486.6 eV) was used to record survey spectra with charge compensation, while high-resolution spectra were recorded without charge compensation. Detection was carried out at an angle of 45° concerning the surface normal, and the analyzed area was 0.5 mm^2^. The curve fitting procedure of the components underlying the carbon (C1s) and oxygen (O1s) peaks was performed using a least-square minimization procedure employing Gaussian–Lorentzian functions and a Shirley-type background. The C1s peaks were referenced at 285 eV (C–C and C–H).

The film compositions were also investigated by attenuated total reflectance-Fourier transform infrared (ATR-FTIR) spectroscopy using a commercial spectrometer (Agilent Cary 660 FTIR, Agilent Technologies, CA, United States), equipped with a deuterated L-Alanine-doped triglycine sulphate (DLa-TGS) detector and a Ge-coated KBr beam splitter. Spectra were recorded in the absorbance mode, and 64 scans were recorded between 500 and 4,000 cm^−1^ with a spectral resolution of 4 cm^−1^.

### 2.4 Trimethoprim Release Kinetic Characterization

Samples of 1.0 ± 0.2 mg (*n* = 3) in weight were obtained from different TMP-loaded film formulations. Samples were placed in 2-ml centrifuge tubes containing 2 ml of PBS (pH 7.4) or MES buffer (pH 5.5). Samples were then incubated at 37°C under agitation (150 rpm). The proportion of 1 mg of film to 2 ml of releasing solutions results in TMP sink condition. At established time points (0, 15, 30, 60, and 180 min and then 1, 3, and 7 days), buffers were collected from the samples and replaced with a fresh buffer. TMP quantification was carried out on the collected aliquots using high-perfomance liquid chromatography (HPLC). A calibration curve was obtained, and the linearity of the concentration ranged from 1 to 100 µg/ml. Aliquots were filtered, transferred to quantification vials, and analyzed with HPLC/UV equipment (Shimadzu, model 20-S, Japan) using X-Bridge C18 150 × 4.6-mm columns, with a particle size of 5 µm. Isocratic chromatography conditions were the mobile-phase acetate buffer 50 mM/acetonitrile (80:20 %v/v) with 0.1% triethylamine, pH 5.9, 1 ml/min flow rate, 40°C column temperature, and 6 min running time. TMP was detected at a wavelength of 254 nm.

### 2.5 Bacteria Stock Preparation

*Escherichia coli* (Gram-negative; ATCC 8937) and *Staphylococcus aureus* (Gram-positive; ATCC 6538) bacteria were used to assess the antibacterial properties of the produced films. All bacteria strains were seeded on freshly prepared, sterile Mueller–Hinton agar in 90-mm Petri dishes and incubated overnight at 37°C in an inverted position. Then, a single colony was picked and incubated in 10 ml of fresh sterile Mueller–Hinton broth overnight at 37° C under shaking (200 rpm). After growth, sterile glycerol at 15% v/v was added for cryopreservation, and bacteria suspension aliquots were frozen at −20°C. The CFU/ml of the stocks after thawing was determined by the log dilution method. In the same way, the bacteria were prepared before assay inoculation.

### 2.6 Minimum Inhibitory Concentration

Trimethoprim MIC against *E. coli* and *S. aureus* was assessed by using the broth dilution method in a 96-well culture plate as described elsewhere (Andrews 2001).

### 2.7 Disk Diffusion Test

The Kirby–Bauer susceptibility test was used in this study (Hudzicki 2009). Briefly, *E. coli* and *S. aureus* bacteria (Gram-negative and Gram-positive) were taken from frozen stocks and thawed to room temperature. Approximately 1 × 10^8^ CFU/ml were spread with a Drigalski spatula onto 15-cm Petri dishes coated with fresh sterile Mueller–Hinton agar. Film samples were cut into a 6-mm-diameter disk (standard NCCLS), sterilized with UV irradiation at 254 nm for 15 min on each side, and then placed on Petri dishes containing the bacteria. Plates were then incubated overnight at 37°C in an inverted position. Paper disks impregnated with antibiotic TMP were used as the positive control. After 18 h of incubation, the diameters of the obtained inhibition zones were measured with a digital pachymeter (NCCLS 2003; Matuschek et al., 2014). Experiments were carried out at least in duplicate.

### 2.8 Released Antibiotic Assay Against *S. aureus*

To test the indirect antimicrobial effects of the developed films, samples of 1.0 ± 0.2 mg (*n* = 3) were inserted in sterile microtubes, immersed in 1 ml of sterile Mueller–Hinton broth, and incubated at room temperature in a laminar flow hood to avoid contamination. At selected time points, that is, 6 h, 1 day, and 3, 7, the solutions (hereafter referred to as eluates) were collected and replaced by 1 ml of fresh sterile Mueller–Hinton broth. All the eluates were kept at −20°C until the experiment.

*S. aureus* bacterial stock suspension was thawed and diluted to a final concentration of 1 × 10^6^ CFU/ml in sterile Mueller–Hinton broth and aliquoted in a 96-well culture plate. Subsequently, 100 µL of the different eluates were added to the wells to give the final volume of 200 µL. Negative controls (blanks) were prepared by adding the inoculum to 100 µL sterile antibiotic-free Mueller–Hinton broth. Positive controls were prepared by adding trimethoprim antibiotic at 5 µg/ml final concentration into the well. Plates were incubated at 35° C under shaking (200 rpm) until optical density at 600 nm (OD_600_) reached 0.6–0.8. The OD600 of the samples was determined and compared to the negative controls. Then, the bacterial survival was calculated using Eq. 1.

$$\%Bacterial\;Survival=\frac{Film\;sample\;OD600}{Blank\;OD600}\times \;100.$$ (1)

Experiments were carried out in triplicate.

### 2.9 Statistical Analysis

InStat ™ software (GraphPad Software, La Jolla, CA, United States) was used to perform the statistical analysis using one-way ANOVA with the Tukey post hoc test. Values of *p* < 0.05 were considered significant. The data shown are mean ± standard error (SE).

## 3 Results and Discussion

The surface composition of the different films, assessed by XPS survey analysis, is given in Table 2. When compared to CS blank films, the amount of carbon is similar and independent from the quercetin percentage and TMP’s presence. It ranges from 62 to 66%, whereas quercetin and TMP carbon theoretical percentages are 68.2 and 66.7%, respectively. The quercetin percentage inside the films does not influence the overall film composition, whereas the presence of TMP leads to changes. In this case, the amount of oxygen is decreased from approximately 30% to 20–22%, whereas the nitrogen contribution is increased from 4–5% to 8–13%.

**TABLE 2:** Surface atomic composition assessed by XPS survey analyses and water contact angle measurements.

Film composition		Atomic composition^a^			WCA (in degree)
—	% Quercetin	%C	%O	%N	
CS	0	62.4 ± 0.2	31.4 ± 0.2	5.4 ± 0.2	104.3 ± 1.0
	Q10	65.5 ± 0.8	28.0 ± 0.6	4.1 ± 0.1	108.2 ± 4.2
	Q20	64.5 ± 1.9	27.7 ± 0.7	3.9 ± 1.0	105.7 ± 3.5
	Q30	63.2 ± 1.5	30.0 ± 1.6	4.1 ± 0.1	95.7 ± 4.0
CS/TMP	0	66.4 ± 0.5	19.4 ± 0.3	13.1 ± 0.1	43.5 ± 2.0
	Q10	62.6 ± 0.2	22.0 ± 0.6	8.0 ± 0.7	52.8 ± 4.3
	Q20	63.9 ± 1.1	22.2 ± 1.0	11.1 ± 0.2	71.7 ± 4.4
	Q30	65.3 ± 1.7	20.4 ± 1.5	13.3 ± 0.2	67.9 ± 1.1
Quercetin		68.2	31.8	-	
TMP		66.7	14.3	19.0	

a Traces of contaminants such as Na, Cl, Ca, and P

**FIGURE 1:**
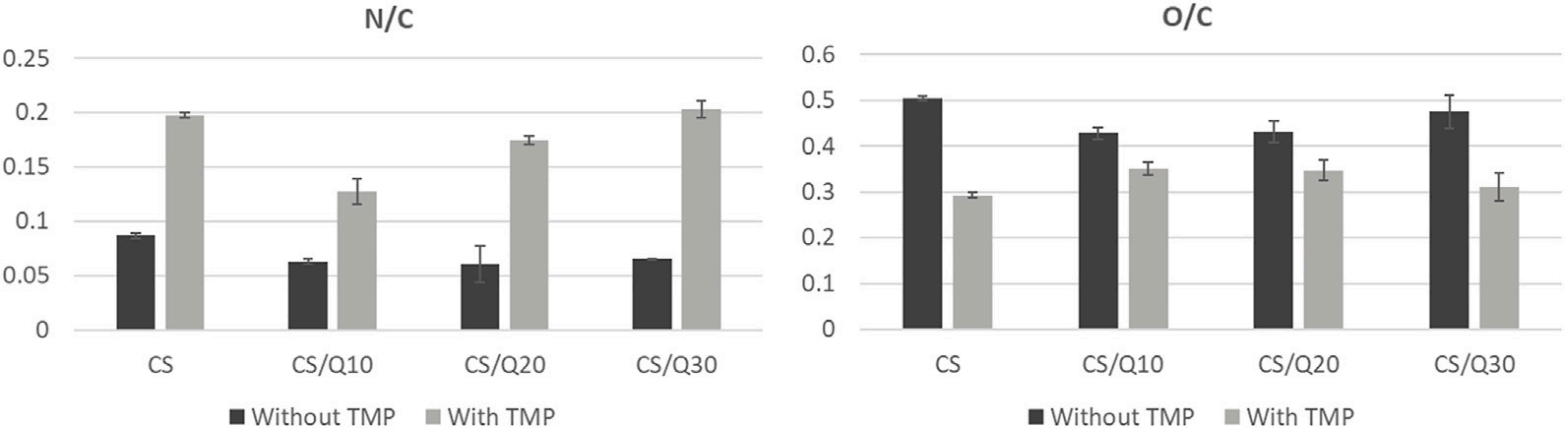
Impact of the quercetin amount and TMP’s presence on N/C and O/C ratios.

As XPS is relative to the atomic percentage, these variations are illustrated in Figure 1 by the N/C and O/C ratios. Once again, the decrease in the O/C ratio due to TMP’s presence is evidenced and does not show any correlation with the quercetin amount. In contrast, N/C ratios increase with the quercetin amount from 0.127 ± 0.012 to 0.203 ± 0.001 for 10 to 30% of quercetin, respectively. TMP films, regardless of the percentage of quercetin, have a higher N/C ratio than those without TMP and thus exhibit a more hydrophilic character. Of note, CS/TMP films without crosslinker exhibit a higher hydrophilic character compared to the films with crosslinker: from 43.5 ± 2.0 up to 67.9 ± 1.1 for CS/TMP and CS/Q30/TMP, respectively. This may be due to the fact that TMP molecules, highly rich in amino groups, known to be hydrophilic, are present in higher numbers on the surface compared to that of crosslinked films. In addition, increasing the amount of quercetin leads to higher water contact angle values, which may be explained by the hydrophobic nature of the aromatic rings present in the quercetin structure. This result is consistent with the literature, where it is clearly stated that the addition of a crosslinker with an alkyl chain, such as glutaraldehyde, leads to more hydrophobic chitosan membranes (Tsai and Wang 2008). Furthermore, Siripatrawan et al. showed that the addition of polyphenolic compounds increased intramolecular interactions, such as hydrogen and covalent interactions, with chitosan reactive groups (OH and NH_2_) (Siripatrawan and Harte 2010). This consequently limits the availability of hydrogen groups to form hydrophilic bonds with water and thus induces a decrease in the chitosan film’s hydrophilicity.

**FIGURE 2:**
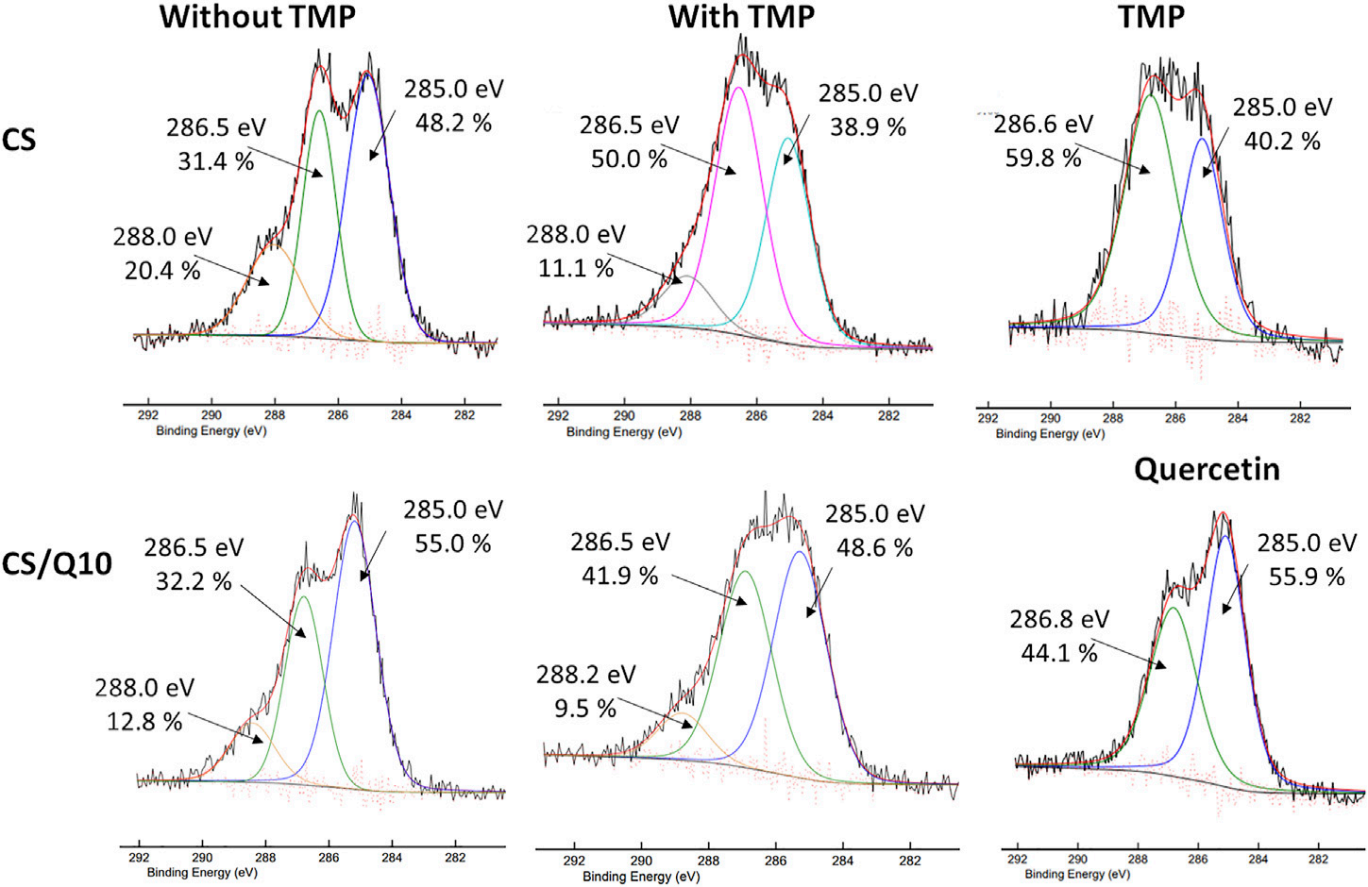
High-resolution C1s XPS spectra of films without and with TMP. Comparison of blank films without quercetin (top) with films containing quercetin 10% (down). TMP and quercetin HR C1s on the right were used for comparison.

The high-resolution XPS spectra of C1s (Figure 2) clearly show the presence of quercetin or TMP molecules on the surface (XPS depth analysis 5 nm), confirming the previous water contact angle measurement results. In fact, the contribution of the band at 286.5 eV associated with C–O/C–N bonds from TMP is higher on CS/TMP films than on the CS/Q10/TMP ones: 50% for CS/TMP, 31.4% for CS, and 41.9% for CS/Q10/TMP films, whereas for TMP molecules, this contribution is 59.8%. The other C1s high-resolution peaks at 285.0 and 288.0 eV are associated with C–C/C–H/C=C bonds, coming from the alkyl and aromatic groups and O–C–O from the acetal moieties of chitosan, respectively. The quercetin presence is also evidenced by an increase in the contribution of the band at 285.0 eV when compared to films without quercetin, as in its structure, quercetin has many aromatic rings.

**FIGURE 3:**
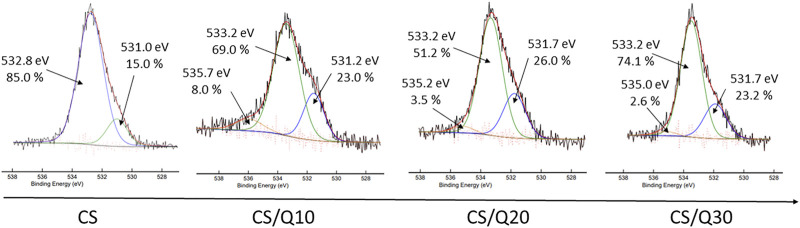
High-resolution O1s XPS spectra of CS/TMP films with different amounts of quercetin, used as a crosslinker, ranging from 0 to 30%.

Based on the C1s spectra, it is not possible to determine whether the crosslinking occurs, thanks to the quercetin presence. Indeed, neither new bands nor significant shifts are noticed, whereas in HR O1s spectra shown in Figure 3, differences can be observed. In particular, a new band between 535.0 and 535.7 eV is associated with quercetin activation. Of note, this band differs from the quercetin spectrum (not shown), which displays two main peaks: one at 531.4 eV, from the pyran moiety, and one at 533.5 eV, with contributions of 12 and 88%, respectively, in accordance with Bourhis et al. (2011). The main one at 533.5 eV is associated with catechol moieties, known for their ability to act as crosslinkers by reacting with nucleophilic groups such as amine and alcohol (Yang et al., 2016) found, for instance, in chitosan’s structure. Briefly, the activation process passes through an activation state under the form of quinones, which appear at a higher binding energy (∼535.5 eV). The quinone contribution decreases when higher quercetin amounts are used, from 8.0 to 2.6% for 10 and 30%, respectively, meaning that there are fewer free quinones. Furthermore, a shift of this band to a lower binding energy is observed, once again meaning that these quinone moieties have reacted with chitosan chains. It may also be noticed that for the 30% quercetin film, the peak centred at 533.2 eV drastically increases to 74.1%, which could be due to unreacted quercetin (in excess).

According to Yang et al. (2016), the reaction between polyphenolic moieties and amino groups can be a Michael-type addition or a Schiff-base reaction. However, both occur in quinones resulting from the previous oxidation of polyphenols. Also, Yang et al. evidenced that the crosslinking chemistry of catechol and amine is both fast and complicated: within 5 minutes, more than 60 products were formed. In addition, polyphenol oxidation, such as dopamine, leads to different quinone forms with different concentrations and reactivity (Quan et al., 2019). Several groups have also demonstrated multiple oxidation structures of quercetin (Sokolová et al., 2012; Stepanic et al., 2015), thus making it difficult to establish a clear mechanism. However, the data on XPS HR C1s and O1s tend to demonstrate that crosslinking occurs and TMP is present on the surface (analysis depth estimated at ∼ 5 nm). However, to have an efficient drug-release system, the bulk composition is crucial as the release of the drug at longer times will arise from a continuous release. Therefore, the bulk composition of the films was assessed by FTIR analyses (depth analysis 1 µm). Due to numerous peaks, only the main ones will be discussed.

**FIGURE 4:**
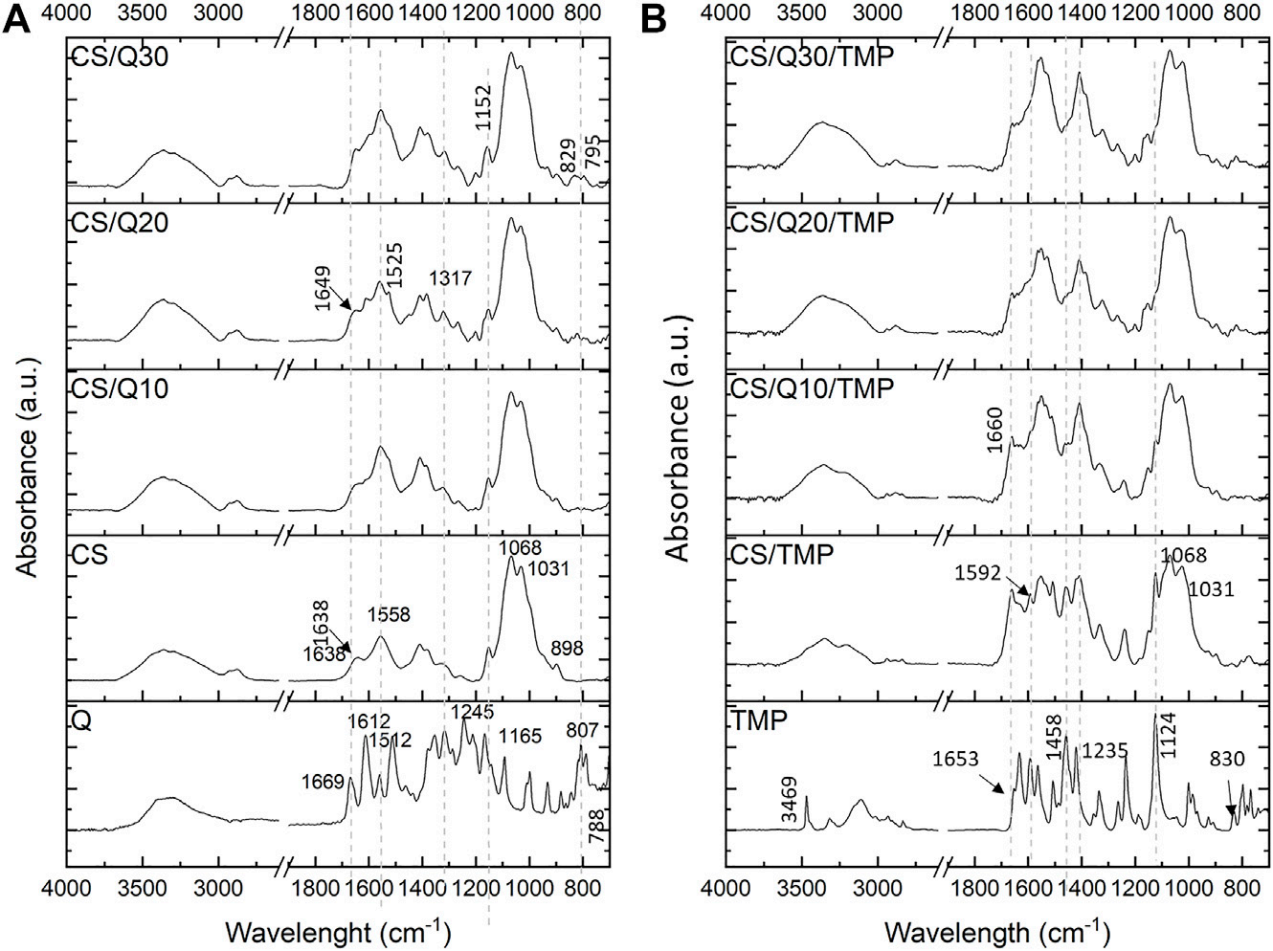
FTIR spectra of all the films **(A)** without TMP and **(B)** with TMP with increasing amount of quercetin (from down to top). Quercetin and TMP spectra were put as references.

The FTIR analyses (Figures 4A) show the expected bands of quercetin, which are the stretching bands of C=O aryl ketone at 1,669 cm^−1^ and of the C=C aromatic ring at 1,612, 1,558, and 1,512 cm^−1^. Bands between 1,245 and 1,165 cm^−1^ are associated with the C–O stretching in the aryl ether ring, phenol, and C–CO–C stretch and bending in the ketone, respectively (Heneczkowski et al., 2001; Catauro et al., 2015). The characteristic bands of chitosan are mainly correlated to the sugar unit at 1,152–1,031 cm^−1^ from C–O–C and C–N stretching and the band at 898 cm^−1^ from the glycosidic bond (Branca et al., 2016). The bands at 1,638 cm^−1^ and 1,558 cm^−1^ are attributed to amide I (C=O) and amide II (NH(CO)) bonds, respectively. In the quercetin-crosslinked chitosan films, the main peaks corresponding to the chitosan sugar unit are unchanged, without factoring in the quercetin amount. This means that the sugar structure of chitosan itself is not affected, as expected. Even though the initial amide bands at 1,638 and 1,558 cm^−1^ are present, they appear slightly modified. Indeed, a shoulder at 1,525 cm^−1^ is detected, and a shift of the amide I band is noticed, from 1,638 cm^−1^ to 1,649 cm^−1^, which may suggest an interaction between chitosan and quercetin. In fact, Sokolová et al. (2011) had noticed a similar shift due to chitosan interaction with quinone moieties. Of interest, the quercetin carbonyl band at 1,669 cm^−1^ is no more visible in the resulting quercetin–chitosan films. It may be assumed that the initial ketone is either shifted due to interactions with chitosan chains or reacted with the surrounding OH/NH_2_ groups since C=N stretching also absorbs from 1,690 to 1,640 cm^−1^. In addition, the intense aromatic CH bending, at 788 and 807 cm^−1^, in the resulting film from quercetin appears very low and shifted to a higher wavelength: 795 and 829 cm^−1^, respectively. This means that there is a loss of quercetin aromaticity, which could be associated with the oxidation of the catechol in quinone, as already noticed in HR O1s spectra. All these observations highlight the interactions of quercetin with chitosan. That said, the introduction of the antibiotic of interest, herein TMP, will increase the complexity of identifying the interactions between the different molecules, as seen in Figure 4B.

The main TMP peaks are assigned as follows: at 3,469 and 3,317 cm^−1^ to N–H stretching vibration, from 1,653 to 1,631 cm^−1^ to amine bending, from 1,458 cm^−1^ to C=C aromatic stretching, at 1,235 and 1,124 cm^−1^ to C–N and C–O stretching, respectively, and at 830 cm^−1^ to C–H bending from the substituted aromatic ring (Ungurean et al., 2013; Da Silva et al., 2016). The TMP peaks are visible on the CS/TMP film, without a clear difference, except a broadening of the area of H bonding in 3,200–3,600 cm^−1^, which may be due to the formation of additional hydrogen bonds as reported by Acharya et al. (2021), whereas on films with quercetin, TMP peaks are less evident, and some band shifts are observed, in particular the band at 1,660 cm^−1^. Indeed, this band was neither detected before on CS/Q films nor on TMP or chitosan films. The contributions of the bands centered at 1,550–1,560 cm^−1^ and 1,408 cm^−1^ become predominant when the amount of quercetin increases. Due to the presence of amino groups in the chemical structure of TMP, the interactions between chitosan, quercetin, and TMP become more complex, which strongly impact the FTIR spectra and complicate the association with bands of specific characteristics.

**FIGURE 5:**
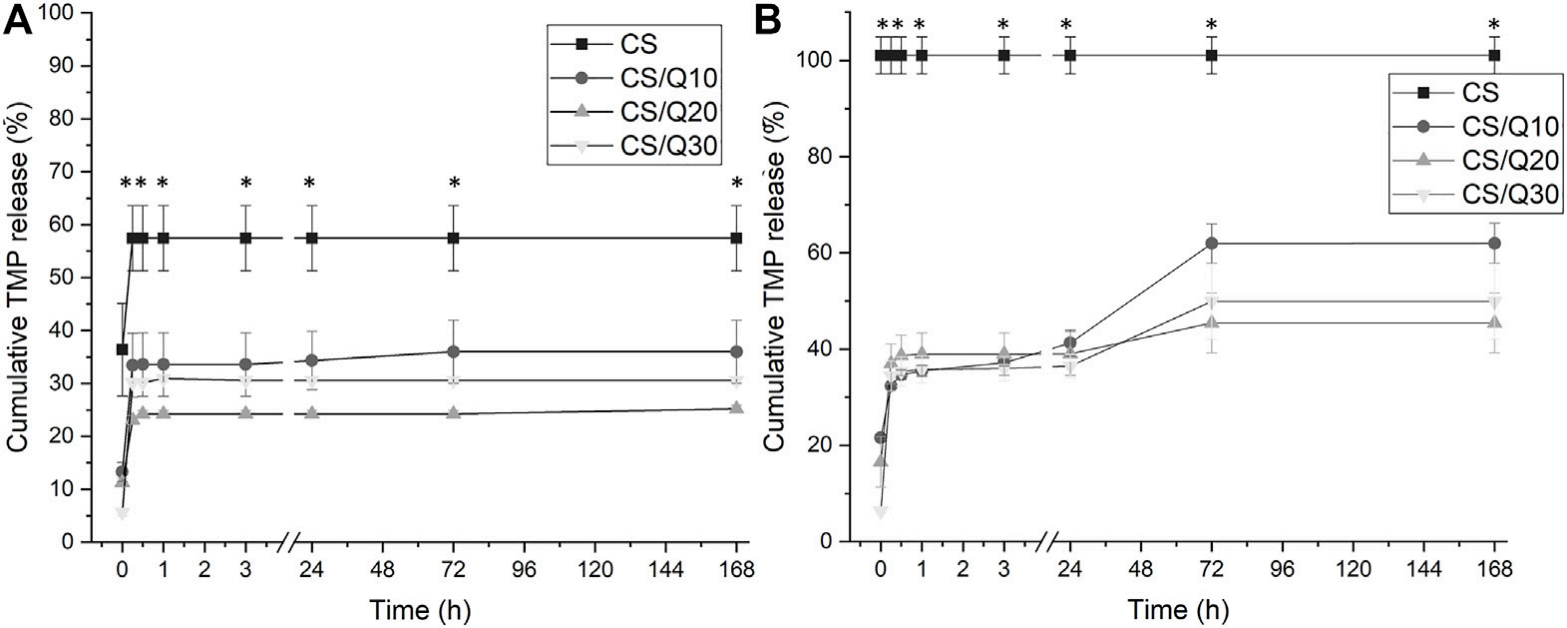
Kinetic release study of TMP from chitosan–quercetin films at different pH: **(A)** pH 7.4 and **(B)** pH 5.5. The graphic shows the mean cumulative release ± SE measured at each time point. *p˂0.001 vs. all the tested conditions at all the tested time points.

TMP release studies were performed in PBS (pH 7.4) and MES (pH 5.5). The two solutions were selected to mimic the pH of human blood and skin/urine, two of the main biomaterial-contacting bodily fluid. The TMP quantification was performed using HPLC/UV-Vis spectrophotometry (ANVISA 2019). The concentration of TMP measured in the release experiments was normalized toward the mass of the films used in the experiments. The cumulative percentage of released TMP is presented in Figure 5.

The quercetin-crosslinked chitosan films demonstrate the capacity of retaining TMP compared to non-crosslinked ones at both the studied pH values. At pH 7.4, the CS/Q10 film releases approximately 30% of the antibiotic-loaded, nearly half of CS/TMP films, within the first hour of incubation, increasing to 35% up to day seven (last time point). CS/Q30 films release more TMP than CS/Q20. This can be due to the excess of non-reticulated quercetin that disturbs antibiotic retention. Nevertheless, all the quercetin-crosslinked samples were able to significantly increase the retention of TMP compared to the chitosan film. Longer exposure time did not result in additional release, indicating a strong interaction of the TMP with the film matrix (Figures 5A). When decreasing the pH to 5.5 (Figures 5B), the non-reticulated film immediately released all the loaded TMP. In contrast, crosslinked films were able to control the release of TMP and its retention in the films with trends similar to the pH 7.4 results: CS/Q20 > CS/Q30 > CS/Q10. Low pH may induce protonation of the chitosan, disrupting the intermolecular interactions with TMP, increasing the antibiotic release. These results show how, with the addition of quercetin, the release is controlled. Similar results were already reported in Wang et al. (2014). Wang et al. showed how quercetin/chitosan conjugate was able to control the release of the loaded drug compared to conventional delivery systems. Moreover, the obtained results indirectly demonstrate the crosslinking effect in the presence of quercetin compared to only chitosan films and that the films release kinetic shows a pH-dependent behavior. This has already been observed in the literature. pH-triggered multilayer films based on chitosan and tannic acid delivery systems have been suggested as controlled release systems under physiological conditions, owing to antiadhesive activity against bacteria (Kumorek et al., 2020). Also, chitosan/quercetin composites have been demonstrated to possess a pH-dependent release kinetic (Mu et al., 2019), confirming the hereby presented results. Furthermore, releasing under acidification is interesting against bacterial biofilms since the biofilm growth of some bacteria such as *Staphylococci* causes a decrease in pH (Liermann et al., 2000; Guimer et al., 2019; Schultze et al., 2020). The decrease in pH values could therefore trigger the release of TMP from the chitosan-quercetin films, acting as a response against bacterial infection.

After physicochemical properties analysis and TMP release kinetic evaluation, antibacterial properties of the films were assessed. To begin with, the minimum inhibitory concentration (MIC) of TMP was measured against *E. coli* and *S. aureus* to evaluate its potency against the strains used in this study (Dafale et al., 2016). MIC analysis results are presented in Table 3.

**TABLE 3:** MIC determination of TMP against the studied bacteria.

Microorganism	TMP MIC (µg/ml) this study	TMP MIC (µg/ml) Eucast database
*E. coli*	0.125	0.06–2.0
*S. aureus*	0.5	0.25–2.0

**FIGURE 6:**
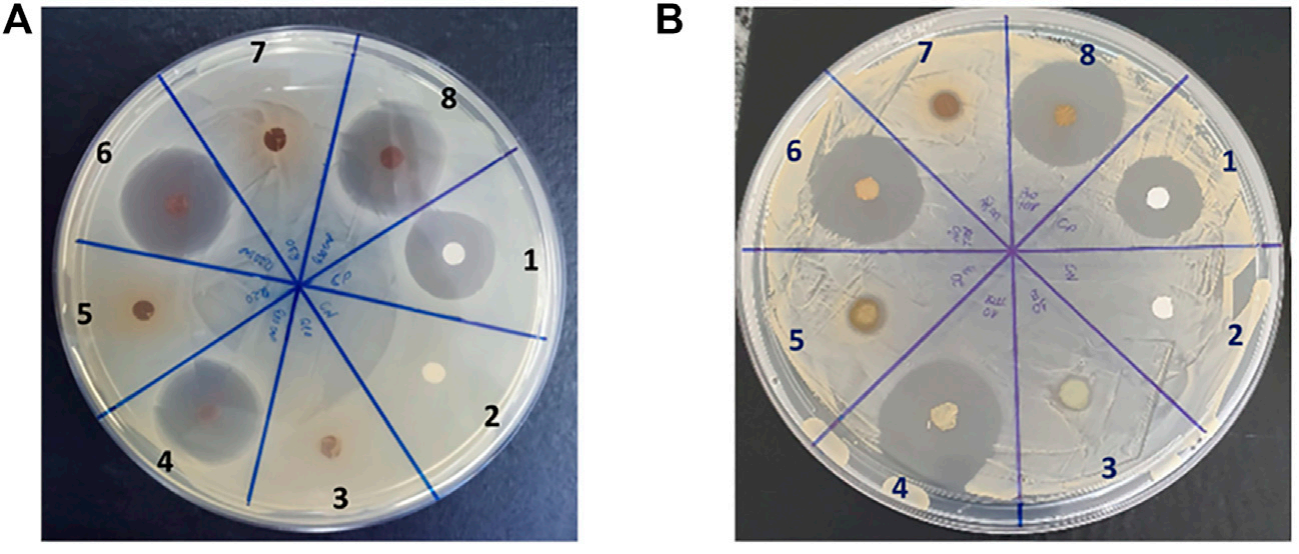
Representative images of disc diffusion (Kirby–Bauer) tests of the chitosan–quercetin crosslinked films loaded with TMP against **(A)** *E. coli* and **(B)** *S. aureus* bacteria. 1) TMP control; 2) negative control; 3) CS/Q10; 4) CS/Q10/TMP10; 5) CS/Q20; 6) CS/Q20/TMP10; 7) CS/Q30; and 8) CS/Q30/TMP10.

As shown, the observed potency values of TMP are in accordance with the range reported in the literature. Afterward, the antibacterial activity of blank and TMP-loaded chitosan films was evaluated by the disk diffusion method (Kirby–Bauer) against *E. coli* and *S. aureus* (Figure 6).

The inhibition halos after 24 h of incubation at 37°C with different film formulations are presented in Table 4.

**TABLE 4:** Disc diffusion results of quercetin-crosslinked chitosan films with different formulations.

	Film composition	Halo (mm) *E.coli*	Halo (mm) *S. aureus*
Blank films			
1	Chitosan control	0	0
2	CS/Q10	0	8.6 ± 0.6
3	CS/Q20	0	10.0 ± 0.6
4	CS/Q30	0	9.7 ± 1.0
TMP-loaded films			
5	Trimethoprim 5 µg	25.2 ± 0.3	29.1 ± 0.9
6	CS/Q10/TMP10	31.5 ± 1.8	37.1 ± 0.9
7	CS/Q20/TMP10	32.2 ± 1.0	38.3 ± 0.2
8	CS/Q30/TMP10	32.0 ± 1.1	38.0 ± 2.1

Chitosan films without crosslinker or antibiotics have been used as a control for antibacterial activity, with no activity. Chitosan–quercetin films without TMP have been evaluated too, and inhibition halos are observed on S. *aureus* plates. This antibacterial activity may be attributed to a partial release of quercetin from the films since chitosan film did not present antibacterial activities. It is known from the literature that quercetin has bactericidal and bacteriostatic properties more prominent on Gram-positive (*S. aureus*) bacteria than on Gram-negative bacteria (*E. coli*) (Amin et al., 2015; Mandal et al., 2017). These observations are in accordance with the presented results. Regarding TMP-loaded chitosan films, paper discs impregnated with 5 µg TMP were used as controls and resulted in a halo of 29.1 mm against *S. aureus* and 25.1 for *E. coli*. Chitosan–quercetin with TMP presented larger halos than the controls. This could have been caused by a synergistic effect between the released TMP and quercetin. In fact, such an effect has already been reported, with quercetin being able to increase the antibacterial efficiency of different antibiotic compounds (Amin et al., 2016). Among the tested antibiotics, TMP’s efficiency was increased by quercetin, both *in vitro* and *in vivo* (Sahyon et al., 2019).

**FIGURE 7:**
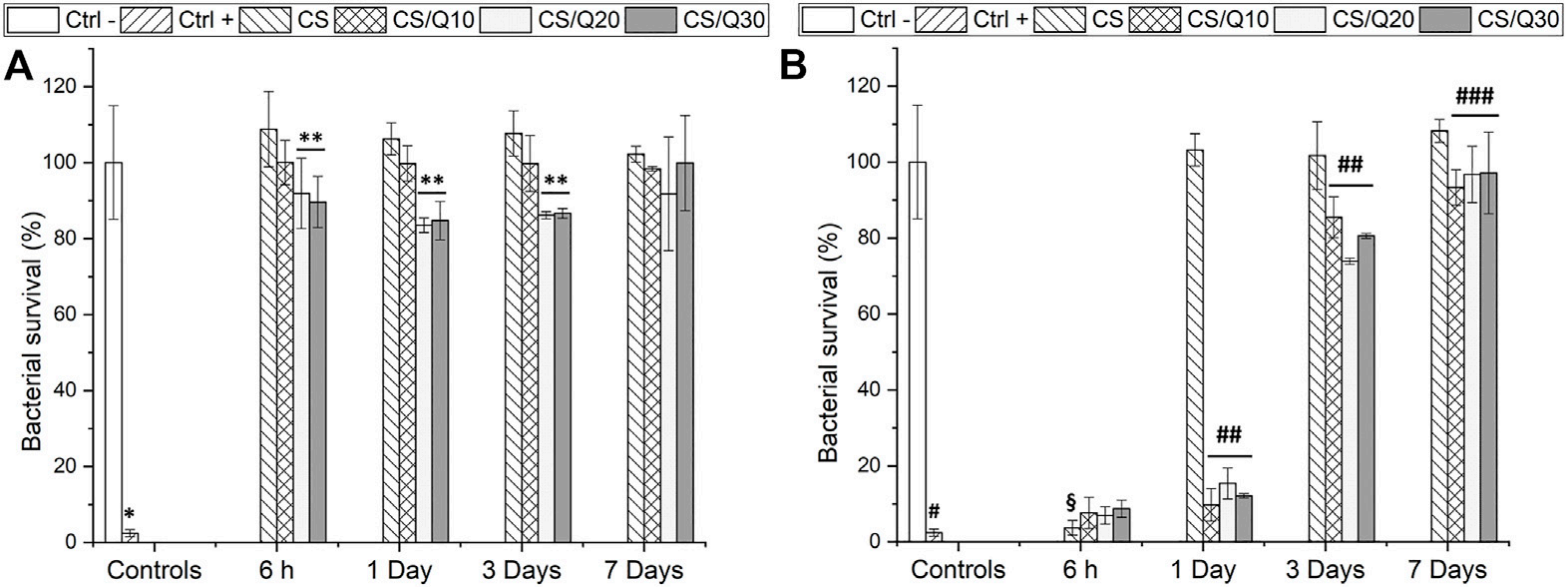
Bacteria survival (*S. aureus*) in MHB media after exposal to the films after 6 h and 1, 3, and 7 days **(A)** Blank quercetin-crosslinked chitosan films and **(B)** TMP-loaded quercetin-crosslinked chitosan films. The graphic shows the mean percentage of bacterial survival ± SD. *p˂0.001 vs. all the tested conditions at all the tested time points; **p˂0.01 vs 6 h and 1 and 3 day CS; #*p*˂0.05 vs. 6 h CS/Q10/TMP, CS/Q20/TMP, and CS/Q30/TMP and *p*˂0.001 vs. all the tested conditions at 1, 3, and 7 days; §*p*˂0.01 vs 6 h CS/Q30/TMP; ##*p*˂0.001 vs. 1 and 3 day CS; ### The graphic shows the mean cumulative release ± SD measured at each time point vs. 7 days CS/Q10/TMP and CS/Q20/TMP and *p*˂0.05 vs. 7 days CS/Q30/TMP.

Afterward, experiments have been carried out to assess the indirect bactericidal activity of the developed films over time (inhibition halo tests done after 1 day). Tests were performed on *S. aureus* due to a higher observed MIC value (Table 3). The trimethoprim MIC value for *S. aureus* in MHB media was used as a threshold for observation since the expected bactericidal activity is due to antibiotic release from the films. Results are shown in Figure 7. The CS/Q20 and CS/Q30 films present mild bactericidal effects toward *S. aureus* for up to 3 days (Figures 7A). Those observations are consistent with our inhibition halo tests and with literature reports of quercetin bactericidal activity (Mandal et al., 2017). Regarding the TMP-loaded films (Figures 7B), the quercetin-crosslinked samples exhibited strong bactericidal properties up to 1 day and mild antibacterial activity after 3 days. This result is consistent with the previously shown TMP release experiments in PBS (Figures 5A) and with the literature, where TMP is known for its efficiency toward *S. aureus* (Zander et al., 2010; Paul et al., 2015). At further time points, TMP is no longer released, therefore showing no bactericidal activity. However, films without quercetin only present bactericidal activity at the first studied time point, when a burst release of TMP occurs. Afterward, with no released antibiotic, no antibacterial effects are noted. Once again, these results demonstrate the effectiveness of quercetin-mediated crosslinking in sustaining the TMP release for a longer time.

## 4 Conclusion

In this study, quercetin-crosslinked chitosan films have been proposed as short-term antibiotic-release systems. It was demonstrated that quercetin reacts with chitosan chains *via* the formation of activated o-quinones, as confirmed by XPS and FTIR experiments. The films containing 20% quercetin (CS/Q20/TMP) showed the highest crosslinking efficiency. The loading of the film with TMP increased the hydrophilic property, and the releasing was pH-dependent. Moreover, the addition of quercetin impacted TMP-controlled release. This effect was further confirmed by the antibacterial test, where the sustained release of TMP was effective up to 3 days for the quercetin-crosslinked films, thus preventing *S. aureus* bacteria growth. Further studies need to be performed to shed light on the biocompatibility of the quercetin–chitosan films. On balance, the TMP-loaded quercetin-crosslinked chitosan films investigated in this work represent an interesting system for the formulation of antimicrobial coatings for the controlled release of bactericidal molecules for short-term applications.

## Data Availability

The raw data supporting the conclusions of this article will be made available by the authors, without undue reservation.

## References

[B1] Acharya V. Ghosh A. Chowdhury A. R. Datta P. (2021). Tannic Acid-Crosslinked Chitosan Matrices Enhance Osteogenic Differentiation and Modulate Epigenetic Status of Cultured Cells over Glutaraldehyde Crosslinking. Soft Mater., 1–12. 10.1080/1539445X.2021.1933032

[B2] Aljadaan S. A. N. Elias R. S. Al-Anssari R. A. (2020). Investigation of the Antioxidant and Antibacterial Activity of Novel Quercetin Derivatives. Biointerface Res. Appl. Chem. 10, 7329–7336. 10.33263/BRIAC106.73297336

[B3] Amin M. U. Khurram M. Khan T. Faidah H. Ullah Shah Z. Ur Rahman S. (2016). Effects of Luteolin and Quercetin in Combination with Some Conventional Antibiotics against Methicillin-Resistant *Staphylococcus A* . Int. J. Mol. Sci. 17, 1947. 10.3390/ijms17111947 PMC5133941 27879665

[B4] Amin M. U. Khurram M. Khattak B. Khan J. (2015). Antibiotic Additive and Synergistic Action of Rutin, Morin and Quercetin against Methicillin Resistant *Staphylococcus A* . BMC Complement. Altern. Med. 15, 59. 10.1186/s12906-015-0580-0 25879586 PMC4364681

[B5] Andrews J. M. (2001). Determination of Minimum Inhibitory Concentrations. J. Antimicrob. Chemother. 48, 5–16. 10.1093/jac/48.suppl_1.5 11420333

[B6] ANVISA (2019). Farmacopeia Brasileira, volume 2. Agência Nacional de Vigilância Sanitária. Brasília: ANVISA.

[B7] Arrieta J. Orrego C. Macchiavello D. Mora N. Delgado P. Giuffré C. (2019). 'Adiós Bacteriemias': A Multi-Country Quality Improvement Collaborative Project to Reduce the Incidence of CLABSI in Latin American ICUs. Int. J. Qual. Heal. Care 31, 704–711. 10.1093/intqhc/mzz051 31198929

[B8] Bai R. Zhang X. Yong H. Wang X. Liu Y. Liu J. (2019). Development and Characterization of Antioxidant Active Packaging and Intelligent Al3+-Sensing Films Based on Carboxymethyl Chitosan and Quercetin. Int. J. Biol. Macromolecules 126, 1074–1084. 10.1016/j.ijbiomac.2018.12.264 30625350

[B9] Bernkop-Schnürch A. Dünnhaupt S. (2012). Chitosan-based Drug Delivery Systems. Eur. J. Pharmaceutics Biopharmaceutics 81, 463–469. 10.1016/j.ejpb.2012.04.007 22561955

[B10] Bourhis K. Blanc S. Mathe C. Dupin J.-C. Vieillescazes C. (2011). Spectroscopic and Chromatographic Analysis of Yellow Flavonoidic Lakes: Quercetin Chromophore. Appl. Clay Sci. 53, 598–607. 10.1016/j.clay.2011.05.009

[B11] Branca C. D'Angelo G. Crupi C. Khouzami K. Rifici S. Ruello G. (2016). Role of the OH and NH Vibrational Groups in Polysaccharide-Nanocomposite Interactions: A FTIR-ATR Study on Chitosan and Chitosan/clay Films | Enhanced Reader. Polymer 99, 614–622. 10.1016/j.polymer.2016.07.086

[B12] Catauro M. Papale F. Bollino F. Piccolella S. Marciano S. Nocera P. (2015). Silica/quercetin Sol-Gel Hybrids as Antioxidant Dental Implant Materials. Sci. Technol. Adv. Mater. 16, 035001. 10.1088/1468-6996/16/3/035001 27877802 PMC5099839

[B14] Da Silva F. E. B. Flores É. M. M. Parisotto G. Müller E. I. Ferrão M. F. (2016). Green Method by Diffuse Reflectance Infrared Spectroscopy and Spectral Region Selection for the Quantification of Sulphamethoxazole and Trimethoprim in Pharmaceutical Formulations. Acad. Bras. Ciênc. 88, 1–15. 10.1590/0001-3765201620150057 26959321

[B15] Dafale N. A. Semwal U. P. Rajput R. K. Singh G. N. (2016). Selection of Appropriate Analytical Tools to Determine the Potency and Bioactivity of Antibiotics and Antibiotic Resistance. J. Pharm. Anal. 6, 207–213. 10.1016/j.jpha.2016.05.006 29403984 PMC5762606

[B16] Dumont C. Nesselrodt D. (2012). Preventing Central Line-Associated Bloodstream Infections CLABSI. Nursing (Lond) 42, 41–46. 10.1097/01.NURSE.0000414623.31647.f5 22561118

[B17] Greco K. V. Francis L. Huang H. Ploeg R. Boccaccini A. R. Ansari T. (2018). Is Quercetin an Alternative Natural Crosslinking Agent to Genipin for Long‐term Dermal Scaffolds Implantation? J. Tissue Eng. Regen. Med. 12 (3), e1716–e1724. 10.1002/term.2338 27717209

[B18] Guimerà X. Moya A. Dorado A. D. Illa X. Villa R. Gabriel D. (2019). A Minimally Invasive Microsensor Specially Designed for Simultaneous Dissolved Oxygen and pH Biofilm Profiling. Sensors 19, 4747. 10.3390/s19214747 PMC6864660 31683828

[B13] Healthcare-Associated Infections (HAIs) (2021). Centers for Disease Control and Prevention. Available at: https://www.cdc.gov/hai/index.html (Accessed February 2, 2022).

[B19] Harwood M. Danielewska-Nikiel B. Borzelleca J. F. Flamm G. W. Williams G. M. Lines T. C. (2007). A Critical Review of the Data Related to the Safety of Quercetin and Lack of Evidence of *In Vivo* Toxicity, Including Lack of Genotoxic/carcinogenic Properties. Food Chem. Toxicol. 45, 2179–2205. 10.1016/j.fct.2007.05.015 17698276

[B20] Health Canada (2018). Evaluation of Healthcare Associated Infection Activities at the Public Health Agency of Canada 2012-13 to 2016-17. Off. Audit Eval. Heal. Canada Public Heal. Agency Canada.

[B21] Heneczkowski M. Kopacz M. Nowak D. Kuźniar A. (2001). Infrared Spectrum Analysis of Some Flavonoids. Acta Pol. Pharm. 58, 415–420. 12197612

[B22] Hudzicki J. (2009). Kirby-Bauer Disk Diffusion Susceptibility Test Protocol. ASM Protoc.

[B23] Kean T. Thanou M. (2010). Biodegradation, Biodistribution and Toxicity of Chitosan. Adv. Drug Deliv. Rev. 62, 3–11. 10.1016/j.addr.2009.09.004 19800377

[B24] Khouri J. Penlidis A. Moresoli C. (2019). Viscoelastic Properties of Crosslinked Chitosan Films. Processes 7, 157. 10.3390/pr7030157

[B25] Kumar A. Kumar A. (2020). The Virtuous Potential of Chitosan Oligosaccharide for Promising Biomedical Applications. J. Mater. Res. 35, 1123–1134. 10.1557/jmr.2020.76

[B26] Kumorek M. Minisy I. M. Krunclová T. Voršiláková M. Venclíková K. Chánová E. M. (2020). pH-Responsive and Antibacterial Properties of Self-Assembled Multilayer Films Based on Chitosan and Tannic Acid. Mater. Sci. Eng. C 109, 110493. 10.1016/j.msec.2019.110493 32228953

[B27] Li X. Sun L. Zhang P. Wang Y. (2021). Novel Approaches to Combat Medical Device-Associated BioFilms. Coatings 11, 294. 10.3390/coatings11030294

[B28] Liermann L. J. Barnes A. S. Kalinowski B. E. Zhou X. Brantley S. L. (2000). Microenvironments of pH in Biofilms Grown on Dissolving Silicate Surfaces. Chem. Geol. 171, 1–16. 10.1016/S0009-2541(00)00202-3

[B29] Mandal S. M. Dias R. O. Franco O. L. (2017). Phenolic Compounds in Antimicrobial Therapy. J. Med. Food 20, 1031–1038. 10.1089/jmf.2017.0017 28661772

[B30] Matuschek E. Brown D. F. J. Kahlmeter G. (2014). Development of the EUCAST Disk Diffusion Antimicrobial Susceptibility Testing Method and its Implementation in Routine Microbiology Laboratories. Clin. Microbiol. Infect. 20, O255–O266. 10.1111/1469-0691.12373 24131428

[B31] Mu Y. Fu Y. Li J. Yu X. Li Y. Wang Y. (2019). Multifunctional Quercetin Conjugated Chitosan Nano-Micelles with P-Gp Inhibition and Permeation Enhancement of Anticancer Drug. Carbohydr. Polym. 203, 10–18. 10.1016/j.carbpol.2018.09.020 30318192

[B32] Nataraj D. Sakkara S. Meghwal M. Reddy N. (2018). Crosslinked Chitosan Films with Controllable Properties for Commercial Applications. Int. J. Biol. Macromolecules 120, 1256–1264. 10.1016/j.ijbiomac.2018.08.187 30176329

[B33] NCCLS (2003). Methods for Dilution Antimicrobial Susceptibility Tests for Bacteria that Grow Aerobically. Sixth Edition. Wayne, Pa: Document M7-A6.

[B34] ODPHP (2021). Healthy People 2030.

[B35] Park S. Y. Marsh K. S. Rhim J. W. (2002). Characteristics of Different Molecular Weight Chitosan Films Affected by the Type of Organic Solvents. J. Food Sci. 67, 194–197. 10.1111/j.1365-2621.2002.tb11382.x

[B36] Paul M. Bishara J. Yahav D. Goldberg E. Neuberger A. Ghanem-Zoubi N. (2015). Trimethoprim-Sulfamethoxazole versus Vancomycin for Severe Infections Caused by Meticillin Resistant *Staphylococcus A*: Randomised Controlled Trial. BMJ 350, h2219. 10.1136/bmj.h2219 25977146 PMC4431679

[B37] Quan W. Y. Hu Z. Liu H. Z. Ouyang Q. Q. Zhang D. Y. Li S. D. (2019). Mussel-Inspired Catechol-Functionalized Hydrogels and Their Medical Applications. Molecules 24 (14), 2586. 10.3390/molecules24142586 PMC6680511 31315269

[B38] Rebelo R. Fernandes M. Fangueiro R. (2017). Biopolymers in Medical Implants: A Brief Review. Proced. Eng. 200, 236–243. 10.1016/j.proeng.2017.07.034

[B39] Sahyon H. A. Ramadan E. N. M. Mashaly M. M. A. (2019). Synergistic Effect of Quercetin in Combination with Sulfamethoxazole as New Antibacterial Agent: *In Vitro* and *In Vivo* Study. Pharm. Chem. J. 53, 803–813. 10.1007/s11094-019-02083-z

[B40] Schultze L. B. Maldonado A. Lussi A. Sculean A. Eick S. (2020). The Impact of the pH Value on Biofilm Formation. Monogr. Oral Sci. 29, 19–29. 10.1159/000510196 33427214

[B41] Scott R. (2009). Centers for Disease Control and Prevention Report. Direct Costs of Healthcare-Associated Infections in U.S. Hospitals and the Benefits of Prevention.

[B42] Siripatrawan U. Harte B. R. (2010). Physical Properties and Antioxidant Activity of an Active Film from Chitosan Incorporated with green tea Extract. Food Hydrocolloids 24, 770–775. 10.1016/j.foodhyd.2010.04.003

[B43] Sokolová R. Degano I. Ramešová Š. Bulíčková J. Hromadová M. Gál M. (2011). The Oxidation Mechanism of the Antioxidant Quercetin in Nonaqueous media. Electrochimica Acta 56, 7421–7427. 10.1016/j.electacta.2011.04.121

[B44] Sokolová R. Ramešová Š. Degano I. Hromadová M. Gál M. Žabka J. (2012). The Oxidation of Natural Flavonoid Quercetin. Chem. Commun. 48, 3433–3435. 10.1039/c2cc18018a 22358256

[B45] Song J. Winkeljann B. Lieleg O. (2020). Biopolymer‐Based Coatings: Promising Strategies to Improve the Biocompatibility and Functionality of Materials Used in Biomedical Engineering. Adv. Mater. Inter. 7, 2000850. 10.1002/admi.202000850

[B46] Souza M. P. Vaz A. F. M. Silva H. D. Cerqueira M. A. Vicente A. A. Carneiro-da-Cunha M. G. (2015). Development and Characterization of an Active Chitosan-Based Film Containing Quercetin. Food Bioproc. Technol 8, 2183–2191. 10.1007/s11947-015-1580-2

[B47] Stærk K. Grønnemose R. B. Palarasah Y. Kolmos H. J. Lund L. Alm M. (2021). A Novel Device-Integrated Drug Delivery System for Local Inhibition of Urinary Tract Infection. Front. Microbiol. 12, 685698. 10.3389/fmicb.2021.685698 34248906 PMC8267894

[B48] Stepanic V. Gasparovic A. Troselj K. Amic D. Zarkovic N. (2015). Selected Attributes of Polyphenols in Targeting Oxidative Stress in Cancer. Curr. Top. Med. Chem. 15, 496–509. 10.2174/1568026615666150209123100 25665579

[B49] Tomaz A. F. de Carvalho S. M. S. Barbosa R. C. Silva S. M. L. Gutierrez M. A. S. de Lima A. G. B. (2018). Ionically Crosslinked Chitosan Membranes Used as Drug Carriers for Cancer Therapy Application. Materials 11, 2051. 10.3390/ma11102051 PMC6213910 30347857

[B50] Tsai H.-S. Wang Y.-Z. (2008). Properties of Hydrophilic Chitosan Network Membranes by Introducing Binary Crosslink Agents. Polym. Bull. 60, 103–113. 10.1007/s00289-007-0846-x

[B51] Ungurean A. Leopold N. David L. Chiş V. (2013). Vibrational Spectroscopic and DFT Study of Trimethoprim. Spectrochimica Acta A: Mol. Biomol. Spectrosc. 102, 52–58. 10.1016/j.saa.2012.10.026 23220519

[B52] VanEpps J. S. Younger J. G. (2016). Implantable Device-Related Infection. Shock 46, 597–608. 10.1097/SHK.0000000000000692 27454373 PMC5110396

[B53] Viola G. M. Rosenblatt J. Raad I. I. (2017). Drug Eluting Antimicrobial Vascular Catheters: Progress and Promise. Adv. Drug Deliv. Rev. 112, 35–47. 10.1016/j.addr.2016.07.011 27496702

[B54] Wang X. Chen Y. Dahmani F. Z. Yin L. Zhou J. Yao J. (2014). Amphiphilic Carboxymethyl Chitosan-Quercetin Conjugate with P-Gp Inhibitory Properties for Oral Delivery of Paclitaxel. Biomaterials 35, 7654–7665. 10.1016/j.biomaterials.2014.05.053 24927684

[B55] Wróbel A. Arciszewska K. Maliszewski D. Drozdowska D. (2020). Trimethoprim and Other Nonclassical Antifolates an Excellent Template for Searching Modifications of Dihydrofolate Reductase Enzyme Inhibitors. J. Antibiot. 73, 5–27. 10.1038/s41429-019-0240-6 PMC7102388 31578455

[B56] Yang J. Saggiomo V. Velders A. H. Cohen Stuart M. A. Kamperman M. (2016). Reaction Pathways in Catechol/Primary Amine Mixtures: A Window on Crosslinking Chemistry. PLoS One 11, e0166490. 10.1371/journal.pone.0166490 27930671 PMC5145154

[B57] Younis M. K. Tareq A. Z. Kamal I. M. (2018). Optimization of Swelling, Drug Loading and Release from Natural Polymer Hydrogels. IOP Conf. Ser. Mater. Sci. Eng. 454, 012017. 10.1088/1757-899X/454/1/012017

[B58] Zander J. Besier S. Faetke S. Saum S. H. Müller V. Wichelhaus T. A. (2010). Antimicrobial Activities of Trimethoprim/Sulfamethoxazole, 5-Iodo-2′-Deoxyuridine and Rifampicin against *Staphylococcus A* . Int. J. Antimicrob. Agents 36, 562–565. 10.1016/j.ijantimicag.2010.08.007 20947313

